# Neonatal mortality in the central districts of Ghana: analysis of community and composition factors

**DOI:** 10.1186/s12889-021-10156-6

**Published:** 2021-01-21

**Authors:** George Adjei, Eugene K. M. Darteh, Obed Ernest A. Nettey, David Teye Doku

**Affiliations:** 1 Department of Community Medicine, University of Cape Coast, Cape Coast, Ghana; 2 Department of Population and Health, University of Cape Coast, Cape Coast, Ghana; 3 University of Ghana, Regional Institute for Population Studies, Accra, Ghana; 4 Kintampo Health Research Centre, P.O. Box 200, Kintampo, Ghana

**Keywords:** Neonatal mortality, Community-level factor, Household-level factor, Individual-level factor, Kintampo health and demographic surveillance system and frailty

## Abstract

**Background:**

Communities and their composition have an impact on neonatal mortality. However, considering the smallest health administrative units as communities and investigating the impact of these communities and their composition on neonatal mortality in Ghana have not been studied. Therefore, this study aimed to investigate the effect of community-, household- and individual-level factors on the risk of neonatal mortality in two districts in Ghana.

**Methods:**

This was a longitudinal study that used the Kintampo Health and Demographic Surveillance System as a platform to select 30,132 neonatal singletons with 634 deaths. Multilevel cox frailty model was used to examine the effect of community-, household- and individual-level factors on the risk of neonatal mortality.

**Results:**

Regarding individual-level factors, neonates born to mothers with previous adverse pregnancy (aHR = 1.38, 95% CI: 1.05–1.83), neonates whose mothers did not receive tetanus toxoid vaccine (aHR = 1.32, 95% CI: 1.08–1.60) and neonates of mothers with Middle, Junior High School or Junior Secondary School education (aHR = 1.30, 95% CI: 1.02–1.65) compared to mothers without formal education, had a higher risk of neonatal mortality. However, female neonates (aHR = 0.61, 95% CI: 0.51–0.73) and neonates whose mother had secondary education or higher (aHR = 0.37, 95% CI: 0.18–0.75) compared to those with no formal education had a lower risk of mortality. Neonates with longer gestation period (aHR = 0.95, 95% CI: 0.94–0.97) and those who were delivered at home (aHR = 0.56, 95% CI: 0.45–0.70), private maternity home (aHR = 0.45, 95% CI: 0.30–0.68) or health centre/clinic (aHR = 0.40, 95% CI: 0.26–0.60) compared to hospital delivery had lower risk of mortality. Regarding the household-level, neonates belonging to third quintile of the household wealth (aHR = 0.70, 95% CI: 0.52–0.94) and neonates belonging to households with crowded sleeping rooms (aHR = 0.91, 95% CI: 0.85–0.97) had lower risk of mortality.

**Conclusion:**

The findings of the study suggest the risk of neonatal mortality at the individual- and household-levels in the Kintampo Districts. Interventions and strategies should be tailored towards the high-risk groups identified in the study.

**Supplementary Information:**

The online version contains supplementary material available at 10.1186/s12889-021-10156-6.

## Background

Over the past two decades, both under-five and neonatal mortality rates have been declining. However, the rates at which neonatal mortality is declining is slower than that of under-five mortality [1]. This phenomenon of slower decline in neonatal mortality has made neonatal mortality relatively stagnant and as a result of that, many regions in the world including sub-Saharan Africa could not achieve Millennium Development Goal (MDG) 4 of reducing under-five mortality by two-thirds [2, 3]. It is projected that if the current trends continue, approximately half of the projected 69 million under-five deaths from 2016 to 2030 will occur during the neonatal period [4]. This makes neonatal mortality a threat to under-five survival and thus the need to study its risk factors.

Sub-Saharan Africa bears the most brunt of neonatal mortality and accounts for 38% of the global neonatal deaths [2, 5]. Over the past two decades, the region has also been experiencing the phenomenon of slower decline in neonatal mortality [6]; making it one of the regions which require huge resources to reducing neonatal mortality significantly. Ghana is no exception with regards to high burden of neonatal mortality. Neonatal mortality accounts for 48% of under-five deaths in Ghana and the rate of neonatal deaths is twice that of post-neonatal deaths [7]. In Ghana, a newborn dies every 15 min [8]. The estimate of neonatal mortality rate in 2014, shows that Ghana’s neonatal mortality rate of 29 per 1000 [7] is greater than Africa’s average of 27 per 1000 [6], and is one of the highest neonatal mortality rates in West Africa [6, 7]. Ghana was not able to achieve MDG 4 due to huge burden of neonatal mortality. This suggests that unless concerted efforts are made, Ghana will not be able to achieve the Sustainable Development Goal (SDG) 3 of reducing under-five and neonatal mortality rates to 25 and 12 per 1000 live births respectively [9].

Between 1990 and 2014, the proportion of neonatal mortality among infants in Ghana increased from 53 to 71% [7]. Within the same period, the proportion of neonatal mortality among under-five deaths almost doubled from 28% in 1990 to 48% in 2014 [7]. Kintampo North Municipality and South District (Hereinafter referred to as Kintampo Districts) located in the middle belt of Ghana are also experiencing a slower decline in neonatal mortality in comparison with post-neonatal mortality [10]. Post-neonatal mortality rate in Kintampo Districts declined significantly from 32 deaths per 1000 live births in 2005 to 21 deaths per 1000 live births in 2009 [10]. On the contrary, neonatal mortality declined slightly from 32 deaths per 1000 live births in 2005 to 31 deaths per 1000 live births in 2009 [10]. This indicates that rates of neonatal and post-neonatal mortality rates were the same in 2005 in the districts but post-neonatal mortality rate reduced significantly in comparison with neonatal mortality rate within the same period. In addition to slower decline in neonatal mortality rates, 42% of neonatal deaths also occur in the communities (Kintampo Health and Demographic Surveillance System (KHDSS) data, 2014). This suggests a high burden of neonatal mortality in the Kintampo districts.

Differences in child health outcomes may be due to differences in community, household or individual characteristics [11, 12]. However, it is widely recognised that socioeconomic attributes, physical structures and the environmental attributes of the community shapes the health outcomes of children in the community [13–24]. Provision of healthcare services, healthcare infrastructure and public services in the community such as sanitation, electricity, water supply, health facilities and availability of healthcare professionals are important community factors that are linked to child differential health outcomes.

The community effect on child and neonatal mortality have been far advanced in theoretical works [23, 25, 26] and empirical literature [12–17, 20–22, 27]. In her theoretical work, Vandresse posited that environmental characteristics such as housing conditions, pollution (air, ground, water and food pollution) and climate of the region in which one resides have effect on infant mortality [23]. The theoretical framework of Mosley and Chen acknowledged the community effect by linking child mortality and socioeconomic determinants of individual, household and community through proximate factors [26]. Regarding empirical literature, several studies [13–16, 18, 20, 22, 27, 28] carried out and a recent work done by Liwin and Houle in Sierra Leone [12] have been able to demonstrate that physical and social structures of the community affect neonatal and child mortality outcomes irrespective of the effect of household- and individual-level factors. These make community factors associated with child and neonatal health risk a key policy tool for the development of public health intervention [11].

Studies on neonatal mortality have been carried out in Ghana [15, 29–32]. However, many of these studies rarely considered the community and its compositional (individual- and household-level) effect on neonatal mortality outcomes. Only one study, to the best of our knowledge, considered community and its compositional effect [15]. However, that study did not consider the health administration sub-districts (smallest units of healthcare system administration in Ghana) as proxies for communities whose operations and planning can be strategically scaled up to provide the necessary and well-focused interventions to reduce neonatal deaths in Ghana. Besides, the design of that study [15] was not longitudinal and therefore lack the temporal sequence needed to establish causality. The temporality provides relevant and reliable evidence for further interventions. This study, therefore, used the Kintampo Health and Demographic Surveillance System (KHDSS) longitudinal dataset to investigate individual-, household- and community (sub-district)-level risk factors of neonatal mortality within the Kintampo Districts.

## Methods

### Study area

This study used longitudinal data from Kintampo Health and Demographic Surveillance System (KHDSS) which covers almost the whole of Kintampo North Municipality and South District. The KHDSS area is sub-divided into 12 sub-districts namely Busuama, Dawadawa, Gulumpe, Kadelso, Kintampo, Kunsu and New Longoro which are located in Kintampo North Municipality while the rest namely Amoma, Anyima, Apesika, Jema and Mansie sub-districts are located in the Kintampo South District [33]. The sub-districts constitute part of the smallest health administrative units of Ghana [34]. The total population and households in the KHDSS area in 2014 were 145,000 and 32,000 respectively. Each district in the KHDSS area has a hospital and other health facilities such as health centres, Community-Based Health Planning and Services (CHPS) compounds and maternity homes that performs family planning services as well as maternal, child and neonatal services. Outreach health services are also performed by CHPS compounds within the communities [35]. Other details of the KHDSS area have been described elsewhere [10].

### Study design

This was a longitudinal study that involved singleton neonates (dead or alive) selected from the KHDSS database between January 2005 and December 2014 to examine the effect of individual-, household- and community-level factors on the risk of neonatal mortality.

### Power calculation

The number of singleton neonates (dead or alive) selected from the KHDSS database between January 2005 and December 2014 was 30,132. Of the 30,132 neonates, 634 were deaths. Therefore, with a sample size of 30,132, neonatal deaths of 634, hazard ratio of 2.0 and a significance level of 0.05, the estimated power of this study was found to be more than 95%.

### Data management

This study used a secondary data which were collected using structured questionnaires by well-trained fieldworkers and entered in the KHDSS database. Hence, all mothers together with their newborns (dead or alive) in the study area (both at home and hospital) between January 2005 and December 2014 were accessed from the KHDSS database. All multiple gestation childbirths (twins, triplets etc.) were identified and removed from the dataset. This was done because they have a high risk of deaths and might therefore not give a true reflection of the strength of relationships between risk factors and neonatal mortality [36]. The community variables were constructed from the dataset using characteristics of the total population within the communities.

### Study variables

The study independent variables were categorised into individual, household and community variables.

#### Individual level variables

The individual variables include sex (male, female), birth order (1, 2–3, ≥4) and gestational age of neonates as well as mothers’ educational level (none, primary, Middle/Junior High School (JHS)/Junior Secondary School (JSS), secondary or above), maternal age at delivery in years (< 20, 20–34, 35+), place of delivery (hospital, health centre/clinic, private maternity home, Traditional Birth Attendant’s (TBA’s) home, at home, other), tetanus toxoid vaccination status (yes, no), gravidity (1, 2–4, ≥ 5) and previous adverse pregnancy (yes, no). Gravidity was the total number of pregnancies mothers of neonates ever had whereas previous adverse pregnancy includes outcomes of previous pregnancies which were ectopic, miscarriage or stillbirth. Tetanus toxoid vaccination status (yes, no) indicates whether a mother received tetanus toxoid vaccine during pregnancy or not. Gestational age of the neonate in weeks was estimated using the reported date of conception and date of delivery.

#### Household level variables

Household level variables considered in this study were crowding and household wealth quintile (poorest, poorer, third, second, wealthiest). Crowding was an index which indicated number of people in a household per sleeping room in a particular year. Household wealth quintile was also an index which was constructed using principal component analysis on household assets and services such as building, car, motorcycle, television, radio, bicycle, farm, table, sleeping mattress, gas cooker, livestock, electricity etc. The procedure for constructing this index has been explained elsewhere [36].

#### Community variables

Community variables considered in this study were ethnic heterogeneity (fractionalization), average distance to the nearest health facility, travel time to water source, poverty, sanitation, water safety and community education. The degree of ethnic heterogeneity (or fractionalisation) at the community level was measured using an index called Ethno-Linguistic Fractionalisation (ELF). ELF is defined as the probability that two individuals randomly selected from an area will belong to two different ethnic groups [37, 38]. ELF method has been explained elsewhere [37, 38]. The theoretical value is zero when there is absolutely no fractionalization (or perfect ethnic homogeneity); and there is absolute fractionalisation (or absolute heterogeneity) when the theoretical value is one. In this study, “Least fractionalised” sub-category were sub-districts with ELF between 0 and 0.19. Sub-districts with ELF between 0.2 and 0.58 were classified as “Moderately fractionalised” whereas sub-districts having ELF in the range 0.59–1 were categorised as “Highly fractionalised”. All the categorisation done was based on literature [37]. Also, distance to the nearest health facility in kilometres was measured using GIS technique. The nearest distance from each household within a particular sub-district in a given year was estimated and the average distance was calculated to represent a particular community’s average nearest distance for a given year. Moreover, the average proportion of households in each community in a given year that used at least 30 min to reach the water source and back was used as proxy for time taken by the community to fetch water. Another independent variable at the community level in this study was poverty level of a particular community. Asset data captured were used to construct wealth quintile for all the households in each sub-district in a given year using PCA. The last two quintiles (poorest and poorer) were classified as poor. The proportion of poor households within the sub-districts was used as a proxy for community poverty level. The proportion of all reproductive age females with secondary education or above in each sub-district within a given year was estimated and used as a proxy for the community educational level.

### Statistical analysis

The analysis of the study involves two parts. Percentages and means were used to describe the distribution of neonatal deaths and general characteristics of neonates for the first part. Means were reported together with the standard deviation whereas percentages were reported together with the frequencies. The second part of the analysis employed multilevel cox proportional hazard model to examine the influence of individual-, household- and community-level factors on neonatal mortality. The multilevel cox proportional hazard model which was constructed hierarchically involved individual-level variables (level 1) nested in household-level variables (level 2) and household-level variables in turn nested in community-level variables (level 3). The individual-level variables were first used to construct the standard cox proportional hazard model (Model I) to assess risk factors of neonatal mortality. The second model (Model II) consist of the individual-level variables in addition to household-level variables with household specific frailty term. Frailty is simply an unobserved effect due to a group (for example household or a community). Hence, household-specific frailty is simply the unobserved effect of the household on neonatal mortality. For example this study did not measure genetic factors so unobserved effect (frailty) at the household level could be due to differential risk in genetic factors. The third model (model III) was constructed by adding the community-level variables with community-specific frailty to the individual- and household-level variables. Scaled Schoenfeld residuals test was used to assess the proportional hazards assumption for all the independent variables used for the multilevel cox regression. This test assessed whether the scaled Schoenfeld residuals of the regression parameters varied with the analysis time [39]. The standard errors of the final model was found to be robust when the variables that violated the assumption were included in the model [40]. Therefore the average effect of these variables over the study period was reported [40]. In addition to scaled Schoenfeld residuals test, Kaplan-Meier observed survival curves and cox predicted curves for each categorical covariate were compared and subsequently assessed for proportional hazards assumption.

Another multilevel cox proportional hazard model was also built by assessing clustering effect at the household and adjusting for individual- and community-level variables (Module IV). The Akaike Information Criterion (AIC) and the Bayesian Information Criterion (BIC) values calculated for each model were used to assess the models goodness of fit. Since lower AIC and BIC values produce better models, Model II should have been the best model. However, Model II did not have the full complement (or full set) of the covariates so Model IV which had the full complement and lower AIC and BIC values as compared to Model III (which also had the full complement) was chosen as the final model. Although Model III and Model IV had the full complement of the covariates, frailty parameters of Models III and IV were communities and households respectively. All statistical tests were two-tailed and *p*-values less than 0.05 were considered as statistically significant. Stata 14.0 and R software were used for the analysis.

## Results

### General characteristics and distribution of neonatal deaths by individual-, household- and community-level characteristics

Table 1 presents the general characteristics of neonates and the distribution of neonatal deaths. Male neonates (51.1%) were slightly more than the female neonates (48.9%). More than half (55.5%) of the neonates had mothers who did not have formal education. Almost 4% (3.7%) belonged to mothers with secondary education or above (Table 1). Approximately 47% of the neonates were delivered at home whereas more than one-third (34.3%) were delivered at the hospital (Table 1). Further details of general characteristics and distribution of neonatal deaths are shown in Table 1.

**Table 1:** General characteristics and deaths by individual-, household- and community-level characteristics

Variable		Neonatal deaths		Total
		Yes	No	
*Individual variables*	n (%)	n (%)	n (%)	N (%)
**Sex**				
Male	15,406 (51.1)	395 (2.6)	15,011 (97.4)	15,406 (100.0)
Female	14,487 (48.9)	239 (1.6)	14,487 (98.4)	14,487 (100.0)
**Total**	29,893 (100.0)			
**Birth order**				
1	7105 (23.6)	181 (2.6)	6924 (97.4)	7105 (100.0)
2–3	10,008 (33.2)	205 (2.1)	9803 (97.9)	10,008 (100.0)
≥ 4	12,994 43.2)	247 (1.9)	12,747 (98.1)	12,994 (100.0)
Total	30,107 (100.0)			
**Educational level**				
None	16,649 (55.5)	317 (1.9)	16,332 (98.1)	16,649 (100.0)
Primary	6562 (21.9)	154 (2.3)	6408 (97.7)	6562 (100.0)
Middle/JHS/JSS	5705 (19.0)	151 (2.6)	5554 (97.4)	5705 (100.0)
Secondary or above	1108 (3.7)	10 (0.9)	1093 (99.1)	1108 (100.0)
**Total**	30,024 (100.0)			
**Tetanus toxoid**				
Yes	20,456 (69.6)	415 (2.0)	20,041 (98.0)	20,456 (100.0)
No	8936 (30.4)	205 (2.3)	8731 (97.7)	8936 (100.0)
**Total**	29,392 (100.0)			
Gravidity				
1	6529 (21.7)	167 (2.6)	6362 (97.4)	6529 (100.0)
2–4	13,998 (46.4)	275 (2.0)	13,723 (98.0)	13,998 (100.0)
≥ 5	9601 (31.9)	192 (2.0)	9409 (98.0)	9601 (100.0)
**Total**	30,128 (100.0)			
**Maternal age at delivery**				
< 20	3715 (12.4)	90 (2.4)	3625 (97.6)	3715 (100.0)
20–34	20,361 (67.8)	414 (2.0)	19,947 (98.0)	20,361 (100.0)
35+	5958 (19.8)	130 (2.2)	5828 (97.8)	5958 (100.0)
**Total**	30,034 (100.0)			
**Previous adverse pregnancies**				
Yes	3512 (11.7)	91 (2.6)	3421 (97.4)	3512 (100.0)
No	26,613 (88.3)	543 (2.0)	26,070 (98.0)	26,613 (100.0)
**Total**	30,125 (100.0)			
**Place of delivery**				
Hospital	10,348 (34.3)	296 (2.9)	10,052 (97.1)	10,348 (100.0)
Health Centre/Clinic	2611 (8.7)	39 (1.5)	2572 (98.5)	2611 (100.0)
Private maternity home	2244 (7.5)	35 (1.6)	2209 (98.4)	2244 (100.0)
TBA’s house	483 (1.6)	12 (2.5)	471 (97.5)	483 (100.0)
At home	14,296 (47.4)	246 (1.7)	14,050 (98.3)	14,296 (100.0)
Other	150 (0.5)	6 (4.0)	144 (96.0)	150 (100.0)
**Total**	30,132 (100.00)			
**Gestational age**	Mean (SD)	Mean (SD)	Mean (SD)	
	38.2 (4.2)	36.3 (5.2)	38.3 (4.2)	25,150 (100.0)
*Household variables*				
		Yes	No	**Total**
	n (%)	n (%)	n (%)	
**Household wealth**				N (%)
Poorest	5703 (18.9)	124 (2.2)	5579 (97.8)	5703 (100.0)
Poorer	5887 (19.6)	123 (2.1)	5764 (97.9)	5887 (100.0)
Third	6099 (20.2)	113 (1.9)	5986 (98.1)	6099 (100.0)
Second	6275 (20.8)	129 (2.1)	6146 (97.9)	6275 (100.0)
Wealthiest	6168 (20.5)	145 (2.3)	6023 (97.7)	6168 (100.0)
**Total**	30,132 (100.0)			
**Crowding**	Mean (SD)	Mean (SD)	Mean (SD)	
	2.8 (1.4)	2.8 (1.4)	3.1 (1.5)	30,062 (100.0)
*Community variables*				
**Ethnic heterogeneity**				
Least fractionalised	879 (2.9)	22 (2.5)	857 (97.5)	879 (100.0)
Moderately fractionalised	2012 (6.7)	33 (1.6)	1979 (98.4)	2012 (100.0)
Highly fractionalised	27,241 (90.4)	579 (2.1)	26,662 (97.9)	27,241 (100.0)
**Total**	30,132 (100.0)			
	Mean (SD)	Mean (SD)	Mean (SD)	
**Average distance to the nearest health facility**	1.6 (0.9)	1.6 (1.0)	1.6 (0.9)	30,132 (100.0)
**Average travel time to water source**	17.8 (14.7)	17.7 (13.8)	17.8 (14.7)	30,132 (100.0)
**Poverty**	41.1 (2.2)	41.2 (2.2)	41.1 (2.1)	30,132 (100.0)
**Sanitation**	28.6 (24.3)	28.2 (24.4)	28.8 (24.3)	30,132 (100.0)
**Water safety**	62.5 (19.9)	63.4 (18.2)	62.5 (19.9)	30,132 (100.0)
**Community education**	3.8 (1.7)	3.9 (1.6)	3.8 (1.7)	30,132 (100.0)

Table 2 presents the scaled Schoenfeld residuals test results for proportional hazards assumptions conducted for each covariate. Graphs depicting plots of Kaplan-Meier observed survival curves and cox predicted curves have also been depicted in the Additional file 1. Additionally, standard errors and robust standard errors for the final model have also been presented in Table 3.

**Table 2:** Proportional hazards assumption test for individual-, household- and community-level variables

Variable	Chi-squared value	Global p-value
Sex	1.44	0.230
Birth order	0.98	0.322
Educational level	0.76	0.384
Tetanus toxoid	0.10	0.753
Gravidity	2.89	0.089
Maternal age at delivery	5.50	0.019^a^
Previous adverse pregnancies	0.02	0.879
Place of delivery	20.35	< 0.001^a^
Gestational age	0.36	0.550
Household wealth	0.00	0.994
Crowding	0.00	0.986
Ethnic heterogeneity	0.00	0.980
Average distance to the nearest health facility	0.30	0.584
Average travel time to water source	0.01	0.908
Poverty	0.23	0.631
Sanitation	0.26	0.611
Water safety	0.02	0.890
Community education	0.19	0.660

^a^Statistically significant and therefore violates the proportional hazards assumption

**Table 3:** Standard and robust standard errors

Variable	Standard error for Model IV	Robust standard error
**Sex**		
Male	–	–
Female	0.092	0.055
**Birth order**		
1	–	–
2–3	0.317	0.376
≥ 4	0.344	0.324
**Educational level**		
None	–	–
Primary	0.118	0.141
Middle/JHS/JSS	0.122	0.161
Secondary or above	0.369	0.137
**Tetanus toxoid**		
Yes	–	–
No	0.101	0.136
**Gravidity**		
1	–	–
2–4	0.323	0.213
≥ 5	0.373	0.270
**Maternal age at delivery**		
< 20	–	–
20–34	0.153	0.165
35+	0.206	0.258
**Previous adverse pregnancies**		
Yes	0.141	0.194
No	–	–
**Place of delivery**		
Hospital	–	–
Health Centre/Clinic	0.209	0.084
Private maternity home	0.209	0.094
TBA’s house	0.390	0.272
At home	0.110	0.064
Other	0.510	0.502
**Gestational age**	0.009	0.008
**Household wealth**		
Poorest	–	–
Poorer	0.144	0.121
Third	0.150	0.104
Second	0.141	0.126
Wealthiest	0.137	0.142
**Crowding**	0.034	0.031
**Ethnic heterogeneity**		
Least fractionalised	–	–
Moderately fractionalised	0.350	0.319
Highly fractionalised	0.300	0.309
**Distance to nearest health facility**	0.062	0.060
**Travel time to water source**	0.005	0.006
**Poverty**	0.022	0.022
**Sanitation**	0.004	0.004
**Water safety**	0.004	0.004
**Community education**	0.058	0.059

### Results for multilevel cox frailty models

Table 4 presents the results for adjusted hazard ratios for model I, II, III and IV with model IV as the final model. In model IV, sex of neonates had a significant impact on neonatal mortality with females having 39% less risk of mortality as compared to males (aHR = 0.61, 95% CI: 0.51–0.73). Maternal level of education had a statistically significant effect on the risk of neonatal mortality (Likelihood ratio *p*-value< 0.01). Neonates whose mothers had Middle, JHS or JSS education, had 30% excess risk of mortality as compared to neonates whose mothers had no formal education (aHR = 1.30, 95% CI: 1.02–1.65). Conversely, neonates whose mothers had secondary or higher level of education had 63% less risk of death in comparison with neonates whose mothers had no formal education (aHR = 0.37, 95% CI: 0.18–0.75). Neonates whose mothers had no tetanus toxoid vaccine during pregnancy had 32% excess risk of mortality as compared to neonates whose mothers received the vaccine during pregnancy (aHR = 1.32, 95% CI: 1.08–1.60). Additionally, neonates whose mothers had previous adverse pregnancy had 38% excess risk of death as compared to those whose mothers had no previous adverse pregnancy (aHR = 1.38, 95% CI: 1.05–1.83). Place of delivery was also a risk factor for neonatal mortality (Likelihood ratio *p*-value< 0.001). In comparison with neonates delivered at the hospital, neonates who were delivered at health centre/clinic (aHR = 0.40, 95% CI: 0.26–0.60), private maternity home (aHR = 0.45, 95% CI: 0.30–0.68) and at home (aHR = 0.56, 95% CI: 0.45–0.70) had on the average 60, 55 and 44% less risk of mortality respectively. Gestational age was found to be a significant risk factor in the model. The risk of neonatal death reduced by 5% for a week increase in the gestational age of neonates (aHR = 0.95, 95% CI: 0.94–0.97). Household wealth of neonates had a significant impact on neonatal mortality (Likelihood ratio *p*-value = 0.035). Neonates in the third household quintile had 30% less risk of mortality as compared to those in the poorest household quintile (aHR = 0.70, 95% CI: 0.52–0.94). In addition, the risk of neonatal mortality reduced by 9% for every unit increase in household crowding index (aHR = 0.91, 95% CI: 0.85–0.97). None of the community level covariates were found to be statistically significant. Moreover, the variance of the household frailty term (θ = 0.52, p-value = 0.48) was not statistically significant. This gave an indication that there was no unobserved heterogeneity at the household level.

**Table 4:**
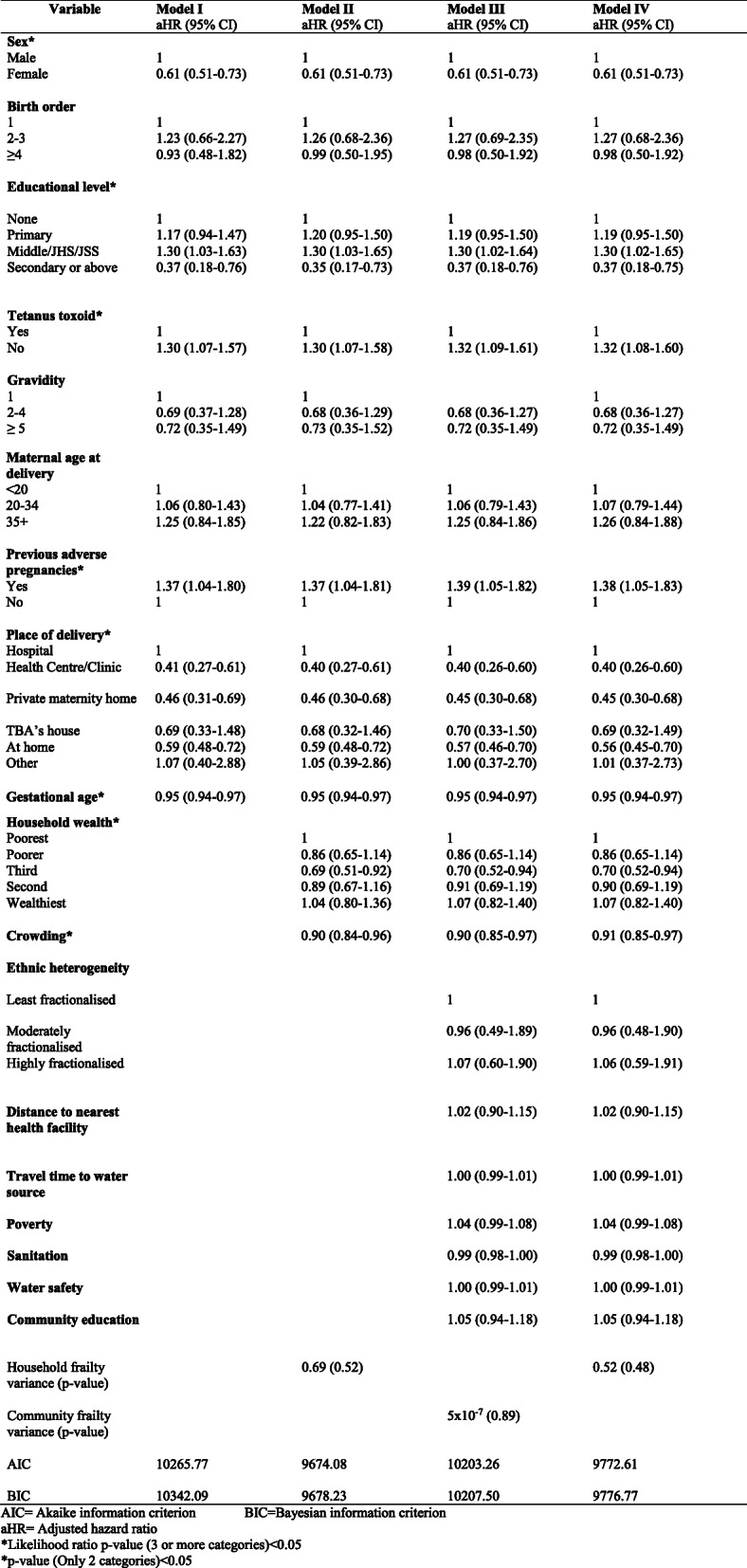
Relationship between neonatal deaths and individual, household and community factors in the Kintampo Districts

## Discussion

Female neonates were found to have lower risk of death than their male counterparts. Findings of several other studies corroborate this finding [18, 21, 41–43]. This finding could be explained by weaker immune system among males attributed to congenital malformation of the urogenital system [20, 21, 44]. On the contrary, Welaga et al. [45] and Kayode et al. [15] have shown that risk of death among male neonates is higher in Ghana but it is not statistically significant. Both studies included both single and multiple births in their analysis and multiple births were found to have a higher risk of neonatal death compared to single births. Perhaps the imbalance effect of multiple births across both sexes attenuated the relationship between risk of neonatal death and sex of neonates.

Maternal level of education also had an impact on neonatal mortality in this study. Neonates whose mothers have middle, JSS or JHS educational level have a higher risk of death than neonates whose mothers do not have formal education. On the contrary, neonates whose mothers have secondary or higher level of education have lower risk of death than those whose mothers do not have formal education. Singh et al. [20] and Mekonnen et al. [18] studies in India and Ethiopia respectively have been able to establish that higher maternal educational level has a lower risk of neonatal mortality. According to literature, higher maternal level of education improves neonatal health outcomes through several channels which includes knowledge and competence of child healthcare; better hygienic practices; more exposure to information and being able to act on this information promptly; able to understand health information better; affordability of healthcare; accessibility of antenatal and prenatal care and the tendency to deliver at the health facility [16, 46, 47]. However, neonates whose mothers had middle, JSS or JHS education have a higher risk of death. Further analysis of the data revealed that, the proportion of neonates whose mothers were teenagers at childbirth and had Middle/JSS/JHS educational level, was 18.9% as compared to 6.9% of neonates whose mothers were teenagers at childbirth and without formal education. This indicates that, proportion of neonates whose mothers are teenagers and have educational level to be Middle/JSS/JHS, is more than twice the proportion of neonates whose mothers are teenagers and without formal education. The higher proportion of teenage mothers among women with Middle/JSS/JHS educational level could be the possible explanation for higher risk of neonatal mortality among this category of women in comparison with women without formal education. The mechanism underlying this phenomenon is that, teenagers are not psychologically and physiologically matured to deliver a baby and these reasons pose a high risk of neonatal mortality among pregnant women who are teenagers [44, 48].

Neonates whose mothers failed to receive tetanus toxoid (TT) injection during pregnancy periods have been also revealed by this study to have higher risk of mortality. This finding is in line with the findings of other studies [18, 20, 49, 50]. In 2015 alone, tetanus caused 19,937 neonatal deaths worldwide [51]. In spite of this number that died from tetanus, coverage of tetanus toxoid vaccination in sub-Saharan Africa is the lowest globally [3]. Coverage is required to increase for the subsequent eradication of this canker. According to 2014 Ghana Health and Demographic Survey report, 78% of births are protected against tetanus but if coverage of tetanus toxoid vaccination is increased and there is huge reduction of pregnant women delivering in an insalubrious environment such as the home, tetanus can be eliminated to avert many neonatal deaths in Ghana.

Neonates whose mothers had previous adverse pregnancies as a result of foetal deaths (miscarriages, stillbirths or ectopic) have an increased likelihood of mortality in comparison with neonates of mothers who did not experience any adverse pregnancies. This finding supported the findings of studies that were carried out in Sudan [52] and Uganda [53] in which mothers with previous adverse pregnancies had 2- and 4-fold increased risk of neonatal mortality. These suggest that previous foetal deaths are linked to the biological deficiency in the reproductive capacity of mothers [23, 44] and this is critical for intervention strategies aimed at reducing neonatal mortality in districts which have similar characteristics as Kintampo Districts.

Paradoxically, neonates belonging to women delivering at home, private maternity home or health centre/clinic have a lower risk of mortality in comparison with neonates whose mothers delivered at the hospital. Kintampo is predominantly rural (65% of the population is rural) and the study’s data showed that, most of the mothers (47.4%) deliver at home in comparison to 34.3% who deliver at the hospital. In total, 65.7% of the mothers deliver at health centres/clinics, private maternity homes, TBAs’ houses, home and other places. Therefore, one of the possible reasons attributed to this finding is the fact that the Kintampo Districts’ hospitals serve as referral hospitals for health centres/clinics, private maternity homes, TBAs’ houses, etc. in the adjoining communities of their catchment area and because of that, severe cases of pregnancies are referred there (hospitals) [54]. The other possible reason may be newborns who are delivered in hospitals and ended up dying at home in the early neonatal period as a result of infections that are high during the early neonatal period [55]. Further analysis of the data revealed that, mothers of neonates who were delivered at the hospital had more previous adverse pregnancy outcomes (13.5%) than mothers (10.2%) of neonates who were delivered at home. This could also be a possible reason for a higher risk of neonatal mortality among neonates delivered at the hospital than at home.

This study investigated the effect of increasing gestational age on the risk of neonatal mortality. Risk of neonatal mortality decreases for a week increase in gestation age and this has been amplified in the literature [56–58]. Findings of several studies in Africa and Asia [48, 57, 59–62] are also in line with this finding of the study but they expressed gestational age in two categories namely preterm (less than 37 weeks of gestation) and term births (37 or more weeks of gestation).

Household wealth which serves as a proxy index for socio-economic status has been shown by this study to have an impact on neonatal mortality. It has been far advanced in the literature that household wealth exerts impact on neonatal mortality through a set of proximate determinants. Hence, the poorest household is more likely to lack proper education, quality healthcare, good nutrition and proper sanitation [19, 63]. In this study neonates belonging to households in the third quintile have less risk of mortality in comparison with neonates in the poorest households. One of the possible reasons may be education since the highest proportion of neonates (23.1%) [not shown] whose mothers had educational level to be secondary or above are those in the third quintile. The other possible reasons may be that, neonates who are in the third quintile have access to good quality healthcare because of the ability of their parents to afford the cost. The lack of good housing quality and environment may also be a possible reason that predisposes neonates in the poorest households to death.

An increase in crowding index is found to reduce the risk of neonatal mortality. This is inconsistent with available evidence which shows that crowding increases the risk of death since that enhances the spread of pathogens directly from one household member to the other [64–66]. However, Fikree (1993) has posited that mortality through the spread of infectious diseases is more likely to occur in the post-neonatal period than the neonatal and the perinatal periods. He further argued that perinatal and neonatal mortality is related more to the maternal environment during pregnancy, labour and delivery [67]. Hence, any association between crowding and the risk of neonatal mortality may operate through psychosocial stress of the mother (which has been established by literature to have an effect on premature labour which is also associated with high risk of perinatal and neonatal mortality) resulting from crowding [67]. Another possible reason is the limitation of the crowding index in which the area of the sleeping rooms for the household members have not been standardised. For example, a household may have many people for a sleeping room but the sleeping room might be quite larger than that of another household which has few people.

Contrary to the findings of other studies, none of the community level factors was found to have a statistically significant effect on neonatal deaths [15, 18, 21]. The possible reason may be the disparity in the operational definition of communities. Unlike the communities of the other studies which are census tracts, survey clusters etc. [11, 13, 15], the communities in this study are health administrative sub-districts in which members in these communities are likely to be educated through activities of CHPS compounds and health centres. Moreover, this study did not have evidence to show that factors at the household (e.g genetic, mothers’ breastfeeding practices etc.) and community (e.g. poor road network, electricity etc.) levels that were not taken into account had statistically significant effect on neonatal mortality.

The study, however, has some strengths and limitations. The use of longitudinal data which has the characteristic of establishing temporality (risk factors preceding the outcome) is considered a key strength of this study. Unlike survey data, children from the same household can be continuously followed up to collect data on common set of risk factors. Also, this study used the multilevel cox proportional hazards model which has a number of advantages. Firstly, the multilevel cox proportional hazard has the ability to assess both the fixed and random effects in a single model [13, 68]. In addition, it disentangles the individual, household and community level effect on neonatal mortality, while controlling for the hierarchical levels in the data at the same time [15, 68, 69]. This is one of the few studies which has taken the hierarchical nature of the data into consideration in investigating the risk of neonatal mortality. Due to frailty term, this method also overcomes the bias due to unobserved heterogeneity at the household and community level [11]. However, this method has a limitation of hazard ratios decaying over time in favour of frailty effect. The degree of decay depends on the frailty variance θ, and when θ is close to zero the hazard ratio regain their usual interpretation [70]. Since θ was close to zero in this study, the degree of decay was minimal. The study, however, has some other limitations. Neonatal deaths are likely to be under-estimated due to the difficulty in the collection of information related to neonatal deaths in developing countries [71, 72]. However, this will be reduced in this study since the study has Community Key Informants who report any events of births and deaths in-between the scheduled household visits. Furthermore, the study has a limitation of some missing values. However, these missing values are minimal for most of the variables considered. Finally, findings of this study cannot be generalised to the whole country but rather to areas in Ghana which have similar characteristics as Kintampo Districts.

## Conclusions

The study has demonstrated risk of neonatal deaths at the individual- and household-levels in the Kintampo Districts. This indicates that risk factors of neonatal mortality in the districts go beyond the individual-level factors. Also, the findings of the study have shown that, even if two newborns are preterm babies, the one with a longer gestation period has a greater chance of survival. Additionally, previous adverse pregnancies of mothers could compromise their biological reproductive mechanism and consequently lead to a higher risk of neonatal mortality. Finally, inequality in household socio-economic status predisposes neonates to death. Multi-sectoral approach such as education, continuum of care and regular health check-ups could be adopted to reduce neonatal mortality in the districts. Interventions and strategies tailored towards the high-risk groups identified in the study could further reduce the risk of neonatal mortality in the districts.

## Supplementary Information

**Additional file 1.**

## Data Availability

The dataset generated and analysed during the current study are not publicly available due to its ownership by the Kintampo Health Research Centre (KHRC). However, the data are available from the corresponding author on reasonable request from KHRC.
