# Supplementary material for: Neonatal mortality in the central districts of Ghana: analysis of community and composition factors
Source: BMC Public Health. 2021 Jan 21;21:173. doi: 10.1186/s12889-021-10156-6 (PMC7819257; doi:10.1186/s12889-021-10156-6)
Supplement: Supplementary file 1 — Additional file 1. [file 12889_2021_10156_MOESM1_ESM.docx]

**Appendix**

**Proportional hazards assumption graphs comparing Kaplan-Meier observed survival with Cox predicted curves**
